# Factors Predicting Time to TSH Normalization and Persistence of TSH Suppression After Total Thyroidectomy for Graves' Disease

**DOI:** 10.3389/fendo.2019.00095

**Published:** 2019-03-01

**Authors:** Rosa Maria Paragliola, Vincenzo Di Donna, Pietro Locantore, Giampaolo Papi, Alfredo Pontecorvi, Salvatore Maria Corsello

**Affiliations:** ^1^Department of Endocrinology, Università Cattolica del Sacro Cuore, Rome, Italy; ^2^Endocrinology Unit, Fondazione Policlinico Universitario “A. Gemelli' – IRCCS, Rome, Italy

**Keywords:** Graves' disease, total thyroidectomy, TSH, levothyroxine, anti TSH receptor autoantibodies

## Abstract

Hyperthyroidism related to Graves' disease is associated with a suppression of TSH values which may persist after surgery in spite of a LT_4_ replacement therapy at non-TSH-suppressing doses. The aim of this retrospective study was to evaluate the time to TSH normalization in a group of patients who underwent total thyroidectomy for Graves' disease receiving a LT_4_ therapy dose regimen based on a previously published nomogram, and to identify possible correlations between the time to normalization of post-operative TSH values and preoperative clinical and biochemical parameters. 276 patients affected by Graves' disease who underwent surgery between 2010 and 2015, were retrospectively evaluated for clinical and biochemical parameters as well as post-surgical LT4 treatment regimen. Of the 276 subjects, 174 had initiated LT4 dosage corresponding to a previously published nomogram. 59 patients were excluded because their LT4 requirement (in mcg/kg/day) changed and deviated from the nomogram during the follow-up period, 15 patients were excluded because their TSH level was >4 mcU/ml during the first biochemical evaluation and 2 patients were excluded because they had low TSH levels potentially related to central hypothyroidism due to concomitant hypopituitarism. Therefore, 98 patients were included in our statistical analysis. TSH and FT4 were evaluated at the first post-operative assessment and during follow up until the normalization of TSH values was achieved, and then included in the analysis. During the first post-operative evaluation 2 months after surgery, 59/98 patients had TSH values in the normal range (0.4 to 4.0 mcU/ml), while 39/98 patients had a TSH value < 0.4 mcU/mL. The persistence of post-operative TSH levels < 0.4 mcU/ml was significantly correlated (*p* = 0.022) with longer duration of the disease. The value of anti-TSH receptor autoantibodies (TrAb) at the diagnosis of hyperthyroidism, significantly correlated (*p* = 0.002) with the time to TSH normalization in the group of patients with TSH < 0.4 mcU/ml at first control. This retrospective analysis confirms that in subjects who have undergone thyroidectomy for Graves' disease, time to normalization of TSH may be prolonged. Hence, the role of TSH as the “gold standard” to assess the appropriate LT_4_ replacement therapy regimen during the initial months following surgery may need to be reconsidered.

## Introduction

Total thyroidectomy is considered a reasonable ablative option for selected patient affected by Graves' disease when medical therapy is unsuccessful and radioiodine ablation is not feasible or it is contraindicated (1). Since total thyroidectomy provides an immediate resolution of the hyperthyroidism, levothyroxine (LT_4_) substitutive therapy must be started immediately after surgery and TSH levels must be assessed at least 6–8 weeks later in order to determine the need for dose-adjustment so to reach and maintain post-operative TSH values within the normal reference range.

Several strategies for the evaluation of the LT_4_ starting dose after surgery for benign thyroid disease have been proposed (2). While body weight represents a relevant factor in the prediction of the LT_4_ starting dose (3), it certainly is not the only parameter. In particular, our recently published nomogram considers other factors such as age and body mass index (BMI) (3), with LT_4_ requirement ranging from 1.8 mcg/kg (for subjects < 40 years and with BMI < 23 kg/m^2^) to 1.4 mcg/kg (for subjects > 55 years and with BMI > 28 kg/m^2^). This proposed nomogram allows to the achievement of “normal” TSH values (i.e., 0.4–4 mcU/mL) in 68% of patients at the first post-surgical evaluation (3).

Post-surgical hypothyroidism for Graves' disease represents a peculiar model. In fact, for a variable time between the disease onset and surgery, the hyperthyroidism causes a suppression of TSH values which may persist after surgery in spite of a LT_4_ replacement therapy aimed at obtaining normal TSH values, as often seen in the clinical practice.

The aim of this retrospective study is to evaluate the time of TSH normalization in patients who underwent total thyroidectomy for Graves' disease and who were treated with LT_4_ therapy based on our previously published nomogram (3), and to evaluate possible correlations between the time of TSH normalization and preoperative clinical and biochemical parameters.

## Materials and Methods

We retrospectively evaluated 276 patients affected by Graves' disease who underwent surgery between 2010 and 2015. Total thyroidectomy has been performed by the same surgeon in our Institution (Catholic University School of Medicine, Rome). All patients underwent pre- and post-surgical clinical and biochemical evaluations in our Institution.

Retrospective analysis of the clinical parameters included:

- gender;
- age at the diagnosis and at the time of surgery;
- duration of disease;
- medium dosage of anti-thyroid drug used for the medical treatment of hyperthyroidism and immediately before surgery;
- final histology;
- body mass index (BMI) [kg/m^2^]
- LT_4_ dosage (expressed in mcg/kg/day) prescribed after surgery and during each biochemical control.

Retrospective analysis of the biochemical parameters included:

- thyroid function test and anti-TSH receptor autoantibodies (TrAb) at the onset of hyperthyroidism and immediately before surgery;
- TSH and FT_4_ at the first evaluation (2 months after surgery);
- for patients who did not reach normal TSH values at the first check, TSH and FT_4_ were further performed about every 4 months until the normalization of TSH.

Thyroid ultrasound to exclude significant remnant thyroid tissue after surgery was performed using a real-time ultrasound (10–12 MHz linear transducer).

Biochemical evaluations were performed in the same laboratory of our institution. TSH, FT_4_, and FT_3_ results were measured by chemiluminescent assays. Analytical sensitivity of TSH, FT_3_, and FT_4_ is < 0.0025 mcU/mL, < 1 pg/ml and < 4 pg/ml, respectively. For TrAb, electrochemiluminescent IMA (Roche Diagnostics, Mannheim, Germany) on Cobas E 8,000 platform was used. Cut-off value suggested by the manufacturer was 1.75 U/L.

We classified patients on the basis of age and BMI and evaluated the dosage of LT_4_ (mcg/kg) prescribed after surgery.

We retrospectively included only 174 patients who after surgery had started LT_4_ which corresponded to the dose calculated according to our previously published nomogram (Table 1). Among these 174 patients:

- 59 were excluded because their LT4 requirement (in mcg/kg/day) changed and deviated from the nomogram during the follow-up period, for example, for significant weight variations or for clinical reasons which suggest to increase or to reduce the posology. In other words, we have considered in the statistical analysis only the patients in which the LT4 requirement remained unchanged over time. In this way, we can reasonably suppose that changes in TSH levels are not related to change in LT4 requirement;
- 15 patients were excluded because their TSH level was > 4 mcU/ml during the first biochemical evaluation;
- 2 patients were excluded because they had low TSH levels potentially related to central hypothyroidism due to concomitant hypopituitarism. In these patients, affected by empty sella and pituitary macroadenoma, pituitary function test revealed a hypopituitarism (TSH, GH and gonadotropin deficiency and panhypopituitarism, respectively).

**Table 1:** Nomogram for the prediction of LT_4_ (mcg/kg/day) starting dose after total thyroidectomy (Adapted by Di Donna et al.(3).

**BMI**			
	≤ 23	23–28	>28
**AGE**			
≤40	1.8	1.7	1.6
40-55	1.7	1.6	1.5
>55	1.6	1.5	1.4

*BMI: kg/m^2^; age: years; LT_4_ dose: mcg/kg/day*.

After these selection criteria, 98/174 patients were included in the statistical analysis. “Normal TSH reference range” was considered between 0.4 and 4 mcU/ml. For patients who had a TSH value in the normal TSH reference range at the first post-operative biochemical evaluation, we did not consider further biochemical data during the follow-up. On the contrary, for patients who had TSH values < 0.4 mcU/ml at the first post-operative biochemical evaluation, we considered the following biochemical data until “normalization” of TSH in the reference range was achieved. It is important to underline that we considered only patients who did not change LT_4_ requirement (mcg/kg/day) during their follow-up period. On the basis of these data, we evaluated the time between surgery and TSH normalization.

### Statistical Analysis

Demographic, clinical and biochemical characteristics at the baseline of the enrolled patients and the results of the post-operative evaluations were described by Mean and Standard Error for continuous variables and by frequencies and percentages for categorical variables.

A multivariate logistic regression analysis was performed to identify predictive factors of lack of TSH normalization at the first biochemical evaluation. Factors included in the analysis as explanatory variables included gender, age at diagnosis, preoperative FT4 values, TrAb values at the diagnosis, time between the onset of disease and surgery, dosage of anti-thyroid drug during the medical treatment, dosage of anti-thyroid drug before surgery, type of anti-thyroid drug and histology.

A multivariate linear regression analysis was then performed in order to study possible associations between independent variables (including gender, age at diagnosis, preoperative FT4 values, TrAb values at the diagnosis, time between the onset of disease and surgery, dosage of anti-thyroid drug during the medical treatment, dosage of anti-thyroid drug before surgery, type of anti-thyroid drug and histology) and the duration of TSH normalization in patients who did not reach “biochemical euthyroidism” at their first control.

All statistical analyses were performed using STATA version 13.1 (Copyright 1985–2013 StataCorp LP, 4905 Lakeway Drive, College Station, Texas 77845 USA) and a *p* < 0.05 was considered statistically significant.

## Results

A total of 98 Caucasian patients (89 female and 9 male) were included in the study. Following the diagnosis of hyperthyroidism, patients were treated with medical therapy for a mean of 35 months before surgery. The most commonly used drug was methimazole (87/98, 94.6%). Final histology showed benign disease in 91/98 patients (92.86%) and an incidentally discovered very low-risk carcinoma in 7/98 patients (7.14%). In all 7 cases, the tumor was a well-differentiated classical papillary carcinoma pT1a Nx and, in consideration of the “low risk,” TSH suppression was not considered necessary.

The daily mean dosage of LT_4_ used in the post-operative phase was 1.67 mcg/kg.

Baseline clinical and biochemical characteristics of enrolled patients are presented in Table 2 and the results of the statistical analyses are described in Table 3.

**Table 2:** Clinical characteristics of the enrolled patients.

	**Mean**	**standard error**
Age at diagnosis (years)	36.13	1.17
Age at surgery (years)	38.84	1.19
Time between the onset of disease and surgery (months)	35.11	3.34
Dose of MTM used during the treatment (mg)	10	0.13
Dose of MTM used immediately before surgery (mg)	7.5	0.16
Dose of PTU used during the treatment (mg)	125	0.11
Dose of PTU used immediately before surgery (mg)	150	0.09
Body mass index at the time of surgery	24.39	0.37
LT4 dose after surgery (mcg/kg/day)	1.67	0.01
TrAb at the diagnosis (U/L)	13.29	1.31
FT4 before surgery (pg/ml)	13.78	0.67
FT3 before surgery (pg/ml)	4.7	0.27

*MTM, methimazole; PTU, propylthiouracil; Anti-TSH receptors autoantibodies normal values are < 1.75 U/L, as suggested by the manufacturer*.

**Table 3:** Factors predicting the lack of TSH normalization at the first biochemical valuation.

	***p*-value**
Gender	0.229
Age at diagnosis	0.589
Preoperative FT4 values	0.248
TrAb values at the diagnosis	0.223
**Time between the onset of disease and surgery**	**0.022**
Dosage of anti-thyroid drug during the medical treatment	0.783
Dosage of anti-thyroid drug before surgery	0.356
Type of anti-thyroid drug	0.283
Histology	0.172

*Bold values indicate a significant correlation*.

During the first post-operative evaluation, 59/98 patients (60.2%) had TSH values in the normal range (between 0.4 and 4.0 mcU/ml), while the remaining 39/98 patients (39.8%) had a TSH value < 0.4 mcU/ml (Table 2). For patients who had a TSH values < 0.4 mcU/ml, we considered the following biochemical data (TSH and FT_4_ every 4 months) until “normalization” of TSH in the reference range was achieved. During the TSH-suppressive phase, patients were not clinically thyrotoxic. It is important to underline that LT_4_ replacement dose in this group of patients is the same established by the nomogram and the final stable dose of LT4 (mcg/kg/day) after TSH normalization is the same used during TSH suppression period. Therefore, the biochemical TSH “normalization” can be considered not dependent by LT_4_ replacement dose changes.

**Figure 1:**
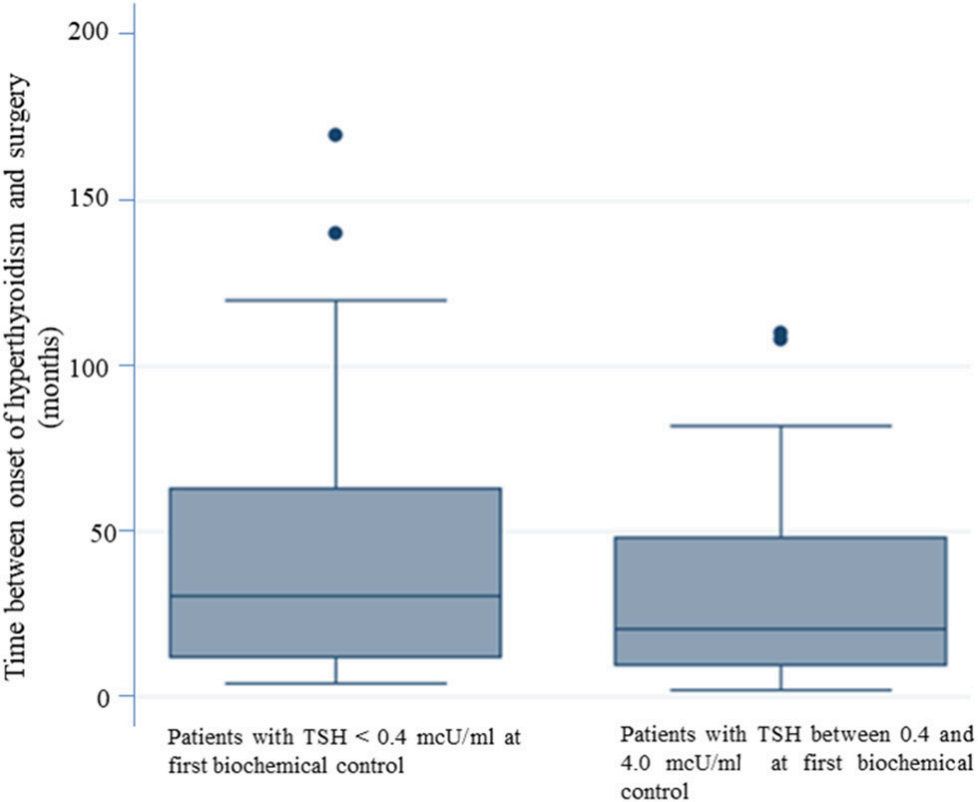
The longer duration of the disease before surgery correlates with the persistence of TSH levels < 0.4 mcU/ml. Patients who reached “biochemical euthyroidism” 2 months after surgery, had a shorter duration of disease between the onset of hyperthyroidism and surgery.

The multivariate logistic regression analysis, aimed at identifying predictive factors of lack of TSH normalization at the first biochemical evaluation, showed that a longer duration of the disease before surgery significantly correlated (*p* = 0.022) with the persistence of TSH levels < 0.4 mcU/ml (Table 3). In patients with normal TSH levels at the first biochemical control the mean time between the onset of disease and surgery was 30.08 months (SE 3.26). For patients with TSH < 0.4 mcU/mL at the first biochemical control, the mean time between the onset of disease and surgery was 42.87 months. Therefore, patients who reached “biochemical euthyroidism” at the time of the first laboratory evaluation (2 months after surgery) had a shorter duration between disease onset and surgery (figure 1).

**Figure 2:**
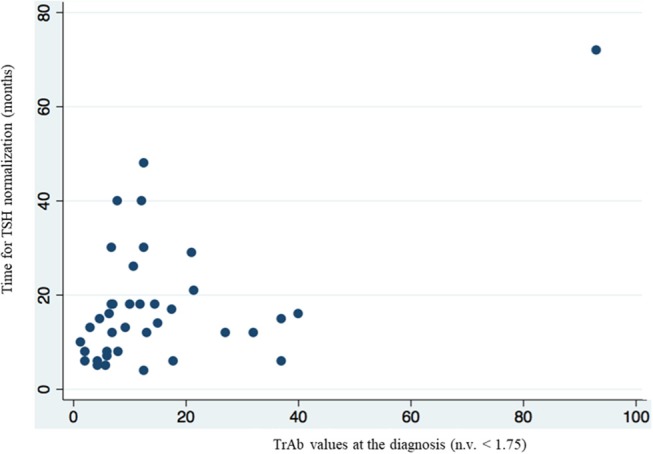
Multivariate linear regression analysis showed a correlation between higher value of TrAb at the diagnosis and longer time of TSH normalization in the group of patients who had TSH < 0.4 mcU/ml at the first biochemical evaluation.

Results by multivariate linear regression also showed that the value of TrAb at the diagnosis of Graves' disease, significantly correlated (*p* = 0.002) with the time to TSH normalization in the group of patients who had TSH < 0.4 mcU/ml at the first control (Table 4, figure 2). The mean value of TrAb at the diagnosis in this group was 14.27 U/L.

**Table 4:** Variables associated with the duration of TSH normalization in patients who did not reach “biochemical euthyroidism” at first post-operative biochemical control.

	***p-*value**
Gender	0.629
Age at diagnosis	0.076
Preoperative FT4 values	0.678
**TrAb values at the diagnosis**	**0.002**
Time between the onset of disease and surgery	0.957
Dosage of anti-thyroid drug during the medical treatment	0.567
Dosage of anti-thyroid drug before surgery	0.691
Type of anti-thyroid drug	0.627
Histology	0.615

*Bold values indicate a significant correlation*.

## Discussion

This retrospective analysis represents the first study in the literature evaluating the time to normalization of TSH values in patients who underwent total thyroidectomy for Graves' disease. Furthermore, the relationship between time to TSH normalization and clinical and biochemical variables has been evaluated. Indeed, while common clinical practice suggests that, using a standard post-surgery LT_4_ dosage, TSH normalizes more slowly in thyroidectomized patients for Graves' disease, to our knowledge no studies have been performed to better characterize this phenomenon and to identify possible predictive factors. Our study confirmed the delay in post-surgical TSH normalization following thyroidectomy due to Graves' disease and an association between this phenomenon and patients' clinical (duration of hyperthyroidism before surgery) and biochemical (TrAb values) pre-surgical parameters was found. About the possible correlation between pre and post-operative TSH levels, in our opinion, the presence of hyperthyroidism and the influence of the antithyroid drugs make pre-operative TSH not reliable. To obtain a model in which TSH normalization is independent of LT_4_ dosage variations, we considered in our statistical analysis only patients who did not modify their LT_4_ requirement (mcg/kg/day) during biochemical follow-up. A reasonable TSH reference range which can be applied to monitor LT_4_ substitutive therapy is between 0.4 and 4 mcU/mL. Indeed, several studies that evaluated “normal” TSH values in the general population reported that the lower TSH limit (2.5th percentile) lies between 0.2 and 0.4 mcU/mL, while upper limits (97.5th percentile) vary between 2.4 and 4.2 mcU/mL, and appear to be related to ethnicity or geographic location (4).

In the present study only about 60% of patients with Graves' disease achieved normal TSH values at the first post-operative biochemical evaluation. Therefore, the performance of the previously published nomogram for the estimation of LT_4_ replacement therapy (3), which was retrospectively evaluated in this group, resulted to be lower than that observed in patients thyroidectomized for other benign diseases. Furthermore, considering that patients with TSH > 4 mcU/mL have been previously excluded from the statistical analysis, all the patients who did not reach normalization of TSH during first post-operative biochemical evaluation, had TSH values < 0.4 mcU/mL.

The longer duration of disease before surgery represents a risk factor for not reaching normalization of TSH at the time of the first biochemical evaluation.

A similar behavior can be observed in patients who, after having undergone surgery for differentiated thyroid cancer and are then being treated with LT_4_ suppressive dose, switch to a substitutive dose regimen. This phenomenon has been described in a recent study which showed long-term pituitary suppression in patients who had been treated with TSH suppressive dose of LT_4_ for a long period following surgery for differentiated thyroid carcinoma (5). In this cohort, even 6 months appear to be not enough for TSH secretion to be restored and to allow the evaluation of thyroid hormone status by measuring serum TSH. The authors suggested that this phenomenon may be due to a long-term suppressive effect exerted by the “excess” of LT_4_, which delays the recovery of the feedback mechanism (5). Similarly, in our group of patients, the persistence of suppressed TSH values can be explained by considering the prolonged TSH suppression induced by the hyperthyroidism.

It is well known, as shown by previous studies, that in the medical treatment of Graves' disease, serum TSH levels may remain low or suppressed for several months to years, despite normalization of FT_3_ and FT_4_ (6). Several mechanisms have been proposed to explain the persistence of TSH suppression after therapy. A systematic review, evaluating 18 articles on this topic, proposed different possible mechanisms (7). Some studies, demonstrated the evidence of a “thyrotroph atrophy,” hypothesizing that this can contribute to the prolonged suppression (8). The histological evaluation of the pituitary performed post-mortem in patients who died during thyrotoxicosis, shows a pronounced decrease or loss of immunoreactivity to TSH, without morphological differences between Graves' disease or Plummer's disease or between genders. However, the loss of pituitary TSH immunoreactivity was found to be reversible in patients with hyperthyroidism after medical treatment, as demonstrated in 16 additionally concurrently studied patients who were in thyrotoxicosis but have been successfully treated with medical therapy and subsequently had normal thyroid function or ypothyroidism (9).

Other studies involved the autocrine and paracrine feedback exerted by TSH at pituitary and hypothalamic levels (10) [ultra-short and short feedback, respectively (11)]. In fact, TSH receptors have been identified on folliculo-stellate cells of the pituitary as well as astroglial cells of the hypothalamus. These receptors are recognized by their specific autoantibodies and are able to downregulate TSH secretion directly or by reducing TRH, independently from thyroid hormone levels (11). The role of immuno-mediated processes, involving the TrAb, has been emphasized by other Authors (12–14). This mechanism probably involves also deiodinase activity, as demonstrated in hyperthyroid sera with TrAb in which the activity of type 2 deiodinase is increased (15).

The time of recovery of TSH has been evaluated in several studies (12, 13, 16–18). Clinical data suggest that TSH recovery is most likely to occur within the first 6 months after treatment, with recovery being achieved in approximately 70% of patients (7). The larger cohort study retrospectively evaluated patients for as long as 30 months, showing that 85.7% of patients have recovered TSH at 30 months (13). In our study, TSH recovery has been reached in 78.9% of patients until 20 months after surgery. The mean time of TSH normalization was about 17 months and normalization was obtained maintaining the same LT_4_ dosage calculated in mcg/kg/day.

Interestingly, the majority of the studies have been conducted in preclinical models, and clinical studies have been limited to patients who reached the remission from Graves' disease by medical therapy or after radioiodine ablation. Instead, data from patients who underwent total thyroidectomy for Graves' disease are lacking. In our opinion, total thyroidectomy is the most accurate model to confirm the delayed recovery of TSH after remission from Graves' disease since it completely and instantaneously removes the confounding effect of any residual thyroid function on post-surgical hormonal assessment. Furthermore, this model allows comparing the trend of TSH values matching them with those of patients who underwent surgery for other disease. To demonstrate this different trend in Graves' disease, we selected only patients who were treated with a LT_4_ dosage targeted to obtain a TSH level in the substitutive dosage reference range.

An interesting finding deduced by our evaluation is the possible role of TrAb in predicting the time of TSH recovery. In fact, the levels of TrAb at the diagnosis of Graves' disease significantly affects the time of TSH normalization during the post-surgical follow-up. Higher TrAb levels at the diagnosis of hyperthyroidism are significantly associated with a longer time of TSH recovery. In our study, all patients with persistence of TSH suppression during the first post-operative evaluations, mean TrAb values at the diagnosis were about 8-fold higher than the normal reference range (14.5 U/L, n.v. < 1.75). There is evidence reported in literature that TrAb play a role in regulating TSH secretion. In fact, Graves' disease is the only cause of hyperthyroidism based on an autoimmune etiology and, with the discovery of TSH receptors within the brain, it has been proposed that TrAb may be involved in the regulation of negative feedback (12). The first observations have been reported in Guinea pigs, in which lower TSH levels have been detected after the injection of IgG from patients with Graves' ophthalmopathy (19). In a clinical study evaluating the thyroid status during treatment with thionamides, higher TrAb values are significantly correlated with suppressed TSH (20), as confirmed also by other Authors(14). An inverse relationship between TrAb and TSH levels may be observed both in euthyroid rats and in euthyroid humans, remarking an active role by autoantibodies in suppressing TSH (13, 14).

A significant limitation of our study, related to the retrospective evaluation, is the lack of TrAb values at the time of TSH normalization. However, the positive correlation between TrAb values at the diagnosis of hyperthyroidism and persistently suppressed TSH levels after surgery, suggest the possibility of a more complex mechanism, involving a persistent activity of TrAb on negative feedback, until several months after surgery. This phenomenon should be analyzed by prospective studies on this topic.

## Conclusions

This retrospective analysis confirms that TSH normalizes slowly in patients who achieve remission for Graves' disease. To our knowledge, this represents the first study on this topic, focused on post-surgery hypothyroidism, while other previous studies were focused on the trend of TSH levels after medical therapy or radioiodine ablation. In our analysis, the persistence of reduced TSH levels is associated with a longer duration of disease before surgery, and the time to TSH normalization correlates with higher TrAb levels at the diagnosis of hyperthyroidism. Aside from possible physio-pathological hypotheses on the regulation of the hypothalamus-pituitary-thyroid axis, the results of our study offer useful suggestions for clinical practice. In fact, in patients affected by Graves' disease, the TSH value alone cannot be considered as the “gold standard” in evaluating the efficacy of the LT_4_ replacement therapy during the first months after surgery. It is, therefore, reasonably to avoid the reduction of LT_4_ therapy in presence of reduced TSH levels when patients present clinically with “euthyrodism.” Furthermore, TSH should be monitored periodically considering that the mean time to TSH normalization in the majority of these patients is about 17 months.

## Ethics Statement

All participants gave their written informed consent to participate to the study.

## Author Contributions

All authors listed have made a substantial, direct and intellectual contribution to the work, and approved it for publication.

### Conflict of Interest Statement

The authors declare that the research was conducted in the absence of any commercial or financial relationships that could be construed as a potential conflict of interest.

## References

[1] Elfenbein DM Schneider DF Havlena J Chen H Sippel RS. Clinical and socioeconomic factors influence treatment decisions in Graves' disease. Ann Surg Oncol. (2015) 22:1196–9. 10.1245/s10434-014-4095-6 25245130 PMC4346454

[2] de Carvalho GA Paz-Filho G Mesa Junior C Graf H. Management of endocrine disease: pitfalls on the replacement therapy for primary and central hypothyroidism in adults. Eur J. Endocrinol. (2018) 178:R231–44. 10.1530/EJE-17-0947 29490937

[3] Di Donna V Santoro MG de Waure C Ricciato MP Paragliola RM Pontecorvi A. A new strategy to estimate levothyroxine requirement after total thyroidectomy for benign thyroid disease. Thyroid. (2014) 24:1759–64. 10.1089/thy.2014.0111 25268754

[4] Spencer CA Hollowell JG Kazarosyan M Braverman LE. National health and nutrition examination survey III thyroid-stimulating hormone (TSH)-thyroperoxidase antibody relationships demonstrate that TSH upper reference limits may be skewed by occult thyroid dysfunction. J Clin Endocrinol Metab. (2007) 92:4236–40. 10.1210/jc.2007-0287 17684054

[5] Kim HI Kim TH Kim H Kim YN Jang HW Kim JH. Delayed TSH recovery after dose adjustment during TSH-suppressive levothyroxine therapy of thyroid cancer. Clin Endocrinol. (2017) 87:286–91. 10.1111/cen.13344 28375573

[6] Ross DS. Serum thyroid-stimulating hormone measurement for assessment of thyroid function and disease. Endocrinol Metab Clin North Am. (2001) 30:245–64. 10.1016/S0889-8529(05)70186-9 11444162

[7] Yu H Farahani P. Thyroid stimulating hormone suppression post-therapy in patients with Graves' disease: a systematic review of pathophysiology and clinical data. Clin Invest Med. (2015) 38:E31–44. 10.25011/cim.v38i1.22574 25864995

[8] Fischer HR Hackeng WH Schopman W Silberbusch J. Analysis of factors in hyperthyroidism, which determine the duration of suppressive treatment before recovery of thyroid stimulating hormone secretion. Clin Endocrinol. (1982) 16:575–85. 10.1111/j.1365-2265.1982.tb03174.x 7105429

[9] Scheithauer BW Kovacs KT Young WF Jr Randall RV. The pituitary gland in hyperthyroidism. Mayo Clin Proc. (1992) 67:22–6. 10.1016/S0025-6196(12)60272-9 1732687

[10] Motta M Sterescu N Piva F Martini L. The participation of “short” feedback mechanisms in the control of ACTH and TSH secretion. Acta Neurol Psychiatr Belg. (1969) 69:501–7. 4316589

[11] Prummel MF Brokken LJ Wiersinga WM. Ultra short-loop feedback control of thyrotropin secretion. Thyroid. (2004) 14:825–9. 10.1089/thy.2004.14.825 15588378

[12] Brokken LJ Wiersinga WM Prummel MF. Thyrotropin receptor autoantibodies are associated with continued thyrotropin suppression in treated euthyroid Graves' disease patients. J Clin Endocrinol Metab. (2003) 88:4135–8. 10.1210/jc.2003-030430 12970276

[13] Chung YJ Lee BW Kim JY Jung JH Min YK Lee MS. Continued suppression of serum TSH level may be attributed to TSH receptor antibody activity as well as the severity of thyrotoxicosis and the time to recovery of thyroid hormone in treated euthyroid Graves' patients. Thyroid. (2006) 16:1251–7. 10.1089/thy.2006.16.1251 17199435

[14] Brokken LJ Scheenhart JW Wiersinga WM Prummel MF. Suppression of serum TSH by Graves' Ig: evidence for a functional pituitary TSH receptor. J Clin Endocrinol Metab. (2001) 86:4814–7. 10.1210/jcem.86.10.7922 11600546

[15] Molnar I Szentmiklosi JA Somogyine-Vari E. Hyperthyroidism in patients with Graves' ophthalmopathy, and thyroidal, skeletal and eye muscle specific type 2 deiodinase enzyme activities. Exp Clin Endocrinol Diab. (2017) 125:514–21. 10.1055/s-0043-113831 28750432

[16] Uy HL Reasner CA Samuels MH. Pattern of recovery of the hypothalamic-pituitary-thyroid axis following radioactive iodine therapy in patients with Graves' disease. Am J Med. (1995) 99:173–9. 10.1016/S0002-9343(99)80137-5 7625422

[17] Woeber KA. Relationship between thyroid stimulating hormone and thyroid stimulating immunoglobulin in Graves' hyperthyroidism. J Endocrinol Invest. (2011) 34:222–4. 10.1007/BF03347070 20855936

[18] Chiovato L Fiore E Vitti P Rocchi R Rago T Dokic D. Outcome of thyroid function in Graves' patients treated with radioiodine: role of thyroid-stimulating and thyrotropin-blocking antibodies and of radioiodine-induced thyroid damage. J Clin Endocrinol Metab. (1998) 83:40–6. 10.1210/jcem.83.1.4492 9435414

[19] Dandona P El Kabir DJ. On the effect of thyrotropin and immunoglobulins related to Graves' disease on thyrotropin synthesis and secretion. Clin Endocrinol. (1978) 9:321–7. 10.1111/j.1365-2265.1978.tb02217.x 581481

[20] Ng ML Tan TT Roslan BA Rajna A Khalid BA. Usefulness and limitations of thyrotropin measurements as a first-line test for follow-up of Graves' patients. Ann Acad Med Singapore. (1993) 22:569–72. 7504901

